# Dietary Polyphenols in Age-Related Macular Degeneration: Protection against Oxidative Stress and Beyond

**DOI:** 10.1155/2019/9682318

**Published:** 2019-03-24

**Authors:** Elzbieta Pawlowska, Joanna Szczepanska, Ali Koskela, Kai Kaarniranta, Janusz Blasiak

**Affiliations:** ^1^Department of Orthodontics, Medical University of Lodz, Pomorska 251, 92-216 Lodz, Poland; ^2^Department of Pediatric Dentistry, Medical University of Lodz, Pomorska 251, 92-216 Lodz, Poland; ^3^Department of Ophthalmology, University of Eastern Finland, Kuopio 70211, Finland; ^4^Department of Ophthalmology, Kuopio University Hospital, Kuopio 70029, Finland; ^5^Department of Molecular Genetics, Faculty of Biology and Environmental Protection, University of Lodz, Pomorska 141/143, 90-236 Lodz, Poland

## Abstract

Age-related macular degeneration (AMD) is a multifactorial disease of the retina featured by degeneration and loss of photoreceptors and retinal pigment epithelium (RPE) cells with oxidative stress playing a role in its pathology. Although systematic reviews do not support the protective role of diet rich in antioxidants against AMD, dietary polyphenols (DPs) have been reported to have beneficial effects on vision. Some of them, such as quercetin and cyanidin-3-glucoside, can directly scavenge reactive oxygen species (ROS) due to the presence of two hydroxyl groups in their B ring structure. Apart from direct ROS scavenging, DPs can lower oxidative stress in several other pathways. Many DPs induce NRF2 (nuclear factor, erythroid 2-like 2) activation and expression of phase II enzymes that are under transcriptional control of this factor. DPs can inhibit A2E photooxidation in RPE cells, which is a source of oxidative stress. Anti-inflammatory action of DPs in RPE cells is associated with regulation of various interleukins and signaling pathways, including IL-6/JAK2 (Janus kinase 2)/STAT3. Some DPs can improve impaired cellular waste clearance, including AMD-specific deficient phagocytosis of the A*β*42 peptide and autophagy.

## 1. Introduction

Dietary polyphenols (phenolics) are numerous and heterogeneous groups of chemicals found in plants and beverages. They can be categorized into several groups according to their chemical structure, origin, potential or actual biological function, and others. Chemically, dietary polyphenols can be divided into the following groups: phenolic acids (benzoic acid and cinnamic acid), flavonoids (isoflavones, neoflavonoids, chalcones, flavones, flavonols, flavanones, flavanonols, flavanols, proanthocyanidins, and anthocyanidins), and polyphenolic amides [1]. Other bioactive polyphenols potentially important for human health are curcumin, resveratrol, ellagic acids and their derivatives, lignans, rosmarinic acid, and others.

**Figure 1:**
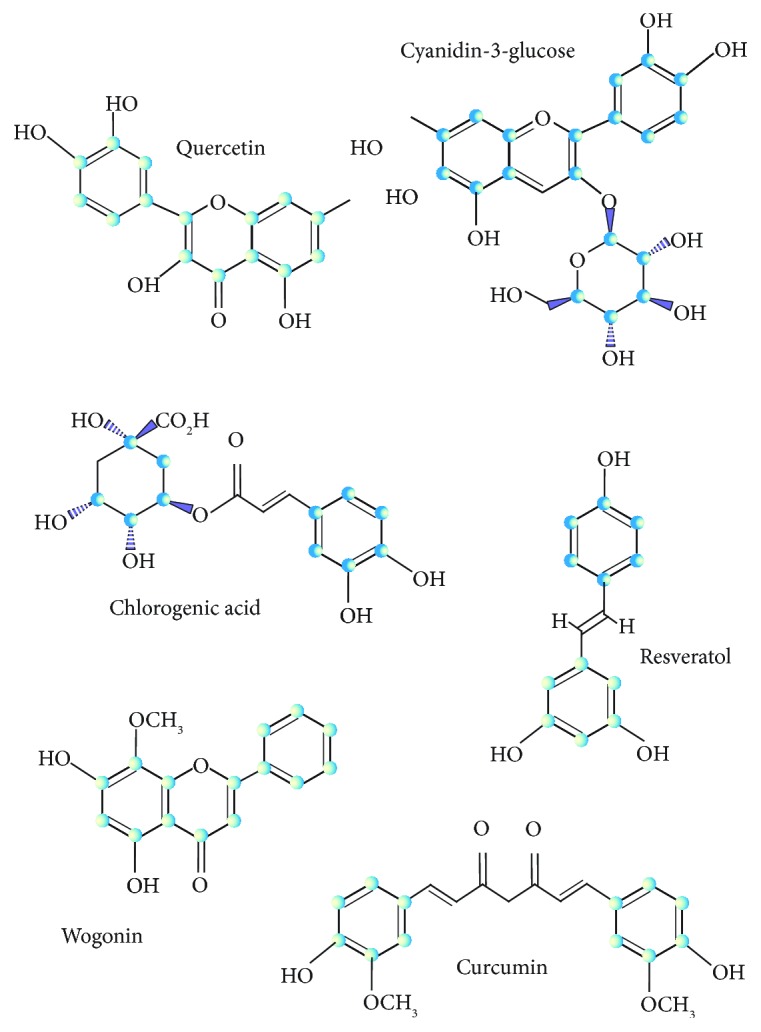
Molecular structure of some dietary polyphenols displaying beneficial properties for human health.

Dietary polyphenols display many activities beneficial for human health—anticancer, anti-inflammatory, antiapoptotic, and antioxidant activities, which are underlined by various mechanisms of their action.  Figure 1 presents some dietary polyphenols, which are reported to be beneficial for human health and presented in this review. Antioxidant activity of these compounds is of special significance for many reasons. First, it can be related to virtually all remaining activities of polyphenolics. Second, oxidative stress is implicated in the pathogenesis of many human diseases. Third, polyphenols, along with some vitamins, reduced glutathione, and other compounds, are members of small molecular weight antioxidants, an important element of cellular antioxidant defense. Although antioxidant activity of polyphenolics in fruits can be higher than that of ascorbic acid, beneficial effects of these compounds for human health are not limited to their antioxidant activity [2].

The beneficial effects of polyphenols on vision is likely the most commonly known health-promoting effect of these compounds [3]. The bioavailability of polyphenolic compounds in the human eye can be adequately high. In a rat in situ perfusion system, the bioavailability of eriodictyol, luteolin, and quercetin was 9, 28, and 20% of the administered dose, respectively [4]. High bioavailability of anthocyanins in ocular tissues of various species after different modes of administration was also reported [5, 6]. These and other reports show that flavonoids, including quercetin and anthocyanins, can cross the blood-retina barrier after oral intake as well as acute intravenous and intraperitoneal administration.

Polyphenols display antiaging properties so they can be considered preventive compounds in age-related human diseases that are usually associated with chronic oxidative stress and accumulation of their products [7]. Therefore, it is reasonable to explore a beneficial potential of dietary polyphenolics in age-related human eye diseases.

## 2. Age-Related Macular Degeneration

Age-related macular degeneration (AMD) is an eye disease mainly affecting people over 65 years that is the major reason of legal blindness and sight loss in the elderly in developed countries. It affects the macula, a highly specialized region of the central retina responsible for fine and color vision. The World Health Organization assesses that 50 million persons suffer from AMD symptoms and 14 million persons are blind or severely visually impaired because of AMD [8]. Therefore, AMD is an important issue in the problem of global vision loss.

**Figure 2:**
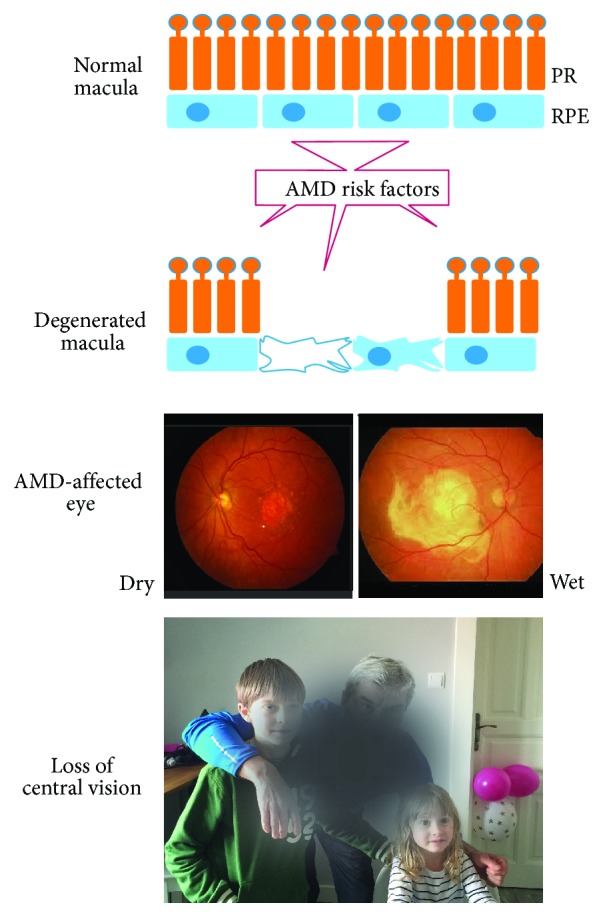
Age-related macular degeneration (AMD). The normal macula located in the central part of the retina contains retinal pigment epithelium (RPE) cells closely interacting with the underlying choroid (not shown) and overlying photoreceptors (PR). Fundus color images of dry and wet AMD. Yellowish spots in the retina called drusen can be seen in dry AMD, whereas wet AMD is featured by the presence of an area of fluid and blood leaked into the retina. AMD impairs the central vision of affected individuals.

In its advanced form, AMD can occur in two distinct forms: dry (atrophic, nonexudative) and wet (exudative), which are shown in Figure 2.

AMD is a complex disease with several risk factors implicated in its pathogenesis. Age is per definition the most serious risk factor for AMD. Mutations in the complement factor H (CFH) are likely the most evidenced genetic change occurring in a fraction of AMD patients [9]. Variability in several loci not involved in the complement pathway is also reported to play a role in AMD pathogenesis. They are the ARMS2 (age-related maculopathy susceptibility 2) gene on chromosome 10 and genes involved in angiogenesis (TGFBR1, VEGFA), the high-density lipoprotein cholesterol pathway (APOE, CETP, and LIPC), immune regulation (PILRB), and others (reviewed in [10]). Epigenetic regulation of gene expression should be also included in studies on AMD pathogenesis, but epigenetic mechanisms in AMD are much less known than their genetic counterparts [11, 12]. Caucasian ethnicity and female sex are frequently associated with AMD occurrence. The environmental/lifestyle factors described as capable of influencing the induction and/or progression of AMD include smoking, obesity, high fat, low antioxidant diet, and exposure to UV and blue light (reviewed in [13]). Some cardiovascular, immunological, and inflammatory biomarkers are reported to be linked to AMD [14–16]. However, except for age, familial history, genetic predisposition, and smoking, other AMD risk factors are still controversial and require further study to confirm their involvement in AMD pathogenesis.

It is commonly accepted that oxidative stress plays an important role in AMD pathogenesis, but the source of oxidative stress in AMD is hardly known. AMD, especially in its wet form, is associated with several changes in retinal vasculature, which can be attributed to aging and intense blood flow in this organ. These changes along with genetic constitution of an individual and environmental/lifestyle factors mainly determine AMD pathogenesis.

## 3. Diet and Oxidative Stress in AMD Pathogenesis

Improper diet can contribute to AMD pathogenesis, and many dietary compounds and supplements were studied in several surveys and trials to assess their protective role in AMD. The main randomized clinical trials on the effect of nutritional compounds on AMD are the Age-Related Eye Disease Study (AREDS); the AREDS2; the Carotenoids in Age-Related Eye Disease Study (CAREDS); the Antioxydants, Lipides Essentiels, Nutrition et Maladies Oculaires (ALIENOR) study, the Taurine, Omega-3 Fatty Acids, Zinc, Antioxidant, Lutein (TOZAL) study; the Blue Mountains Eye Study; the Nutritional AMD Treatment 2 (NAT2) study, the Melbourne Collaborative Cohort Study, and others (reviewed in [17]). Moreover, several meta-analyses on the role of diet in AMD have been performed. However, there is no general agreement on dietary recommendations to protect against AMD induction and progression [18]. Some of clinical trials and meta-analyses did not take into account the genetic constitution of the patients, which may be a source of differences in results of these studies. The AREDS2 recommends a diet supplementation with vitamins C and E, zinc, copper, lutein, and zeaxanthin (AREDS diet, AREDS 2 formula).

Many AMD risk factors, including aging, smoking, UV and blue light exposure, chronic inflammation, and improper diet, can be related to oxidative stress, but it is not known whether oxidative stress associated with AMD belongs to the reasons or consequences of the disease or both. In any case, reduction of the stress can be important in both prevention and therapy of AMD.

**Figure 3:**
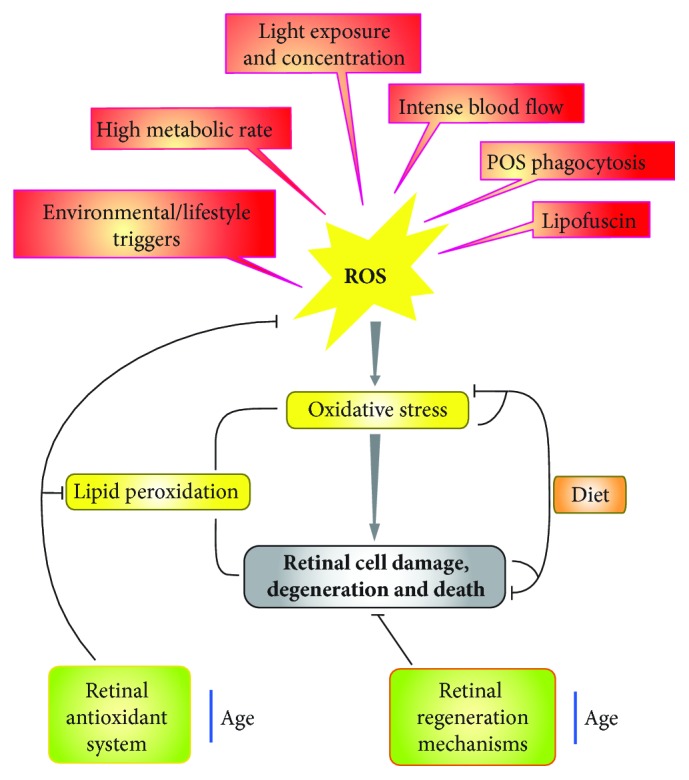
Oxidative events occurring in the retina and their contribution to degeneration and death of retinal cells, an essential feature of age-related macular degeneration. High metabolic rate, intense blood flow in the retina, and its massive light exposure are physiological sources of reactive oxygen species (ROS). Phagocytosis of photoreceptor outer segments (POS) and accumulation of lipofuscin, a protein/lipid mixture, lead to further ROS production. Environmental and lifestyle factors, including additional exposure to blue light, smoking, and diet, potentiate ROS production. However, some nutritional compounds can inhibit oxidative stress associated with ROS overproduction and ameliorate retinal damage. Retinal antioxidant defense can directly inhibit oxidative stress and its intermediates, including lipid peroxidation, but it decreases with age; limited regenerative and renewal mechanisms in the retina also decline with age.

The retina is characterized by the highest rate of metabolism among all tissues of the human body, high oxygen demand, and high vulnerability to oxidative stress [19]. Photoreceptor membranes contain a high fraction of polyunsaturated fatty acids (PUFAs), which are a source of ROS. Chronic exposure to light also results in ROS production. Retinal pigment epithelium (RPE) cells are essential for phototransduction as they phagocytose aged tips of photoreceptor outer segments (POS). POS are loaded phospholipid membrane discs, and they are internalized with the involvement of CD36 (cluster of differentiation 36) and MerTK (Mer tyrosine kinase) with their subsequent autophagic degradation [20]. This process produces reactive intermediates, which along with nondegraded POS and other waste materials can accumulate in the RPE as lipofuscin [21, 22]. This material contains a complex mixture of bisretinoid fluorophores causing fundus autofluorescence [23]. The main fluorophore is A2E (N-retinylidene-N-retinylethanolamin), a pyridinium bisretinoid. When A2E is exposed to blue light, it undergoes photooxidation resulting in ROS production and lipid peroxidation. Oxidative processes in the retina, especially those occurring in the aging macula, can contribute to AMD pathogenesis (Figure 3). Almost all AMD environmental risk factors are associated with ROS overproduction. Aging, the major AMD risk factor, is associated with excess in ROS production in various pathways, including reduced levels of antioxidants and antioxidant enzymes and accumulation of damages to mitochondrial DNA (mtDNA). The macula concentrates light and displays high metabolic activity and high oxygen consumption associated with intense blood flow. The retina has a limited mechanism to regenerate and repair. Retinal pigment epithelium cells, which are nonneural cells of the retina, do not proliferate due to volume constraints. The retina has neither resident stem cells which could produce progenitors to replace degenerated/dead retinal cells nor permanently proliferating cells. When oxidative damage affects the inner part of the retina, including the macula, cells from the outside reinitiate the cell cycle and proliferate to replace their degenerated counterparts. Therefore, the ability of the retina to self-repair and regenerate is restricted. However, DNA repair in RPE cells may occur in a similar fashion as in other human somatic cells, which is of considerable importance as damage to mtDNA can lead to malfunctioning of electron transport chain, resulting in ROS overproduction, which can further damage mtDNA—a classical viscous cycle.

The retina has multiple elements of antioxidant defense and vitamins C and E, and the carotenoids lutein and zeaxanthin are frequently considered the most important [17]. The highest concentration of carotenoids is in the plexiform area of the macula with zeaxanthin dominating in the central fovea and lutein preferentially distributed in the peripheral region of the retina [24]. Both carotenoids are present in the outer segment of rods and cones [25].

Oxidative stress can be potentiated by improper diet. Omega-3 fatty acid docosahexaenoic acid (DHA) is a molecule with six double bonds, prone to the action of ROS, resulting in lipid peroxidation, whose products can damage proteins, nucleic acids, and other cellular components. It is estimated that DHA constitutes about 60% of all lipids in the membrane of photoreceptor cells [17]. DHA can be synthesized from eicosapentaenoic acid (EPA), whose main source in humans is the diet [26]. Both DHA and EPA can induce several beneficial effects in the retina, and their insufficiency in that organ can result in retinal disorders. These omega-3 fatty acids are supplied to photoreceptor membranes by RPE cells. Any imbalance in photoreceptor lipids can contribute to drusen formation in the RPE and sub-RPE layers [27].

Currently, there is no remedy for dry AMD, and wet AMD is treated with moderate successes with vascular endothelial growth factor (VEGF) inhibitors. However, high doses of dietary antioxidant supplements were reported to slow the progression of an intermediate-to-advanced form of the disease [28, 29]. Therefore, documented dietary intervention in AMD is mainly limited to antioxidant activity of dietary compounds and supplements. However, as AMD is a multifactorial disease, risk factor not directly associated with the diet should be taken into account in projecting and analyzing dietary interventions, which was clearly shown in the Carotenoids in Age-Related Eye Disease Study (CAREDS) [30]. Polyphenolic-rich diet has beneficial effects for many AMD-related risk factors, such as obesity, hypertension, and hypercholesterolemia, and healthy diet has been shown to attenuate genetic obesity risk [31]. Polyphenol intake with a Mediterranean diet decreases inflammatory biomarkers, hypertension, and lipid profile related to atherosclerosis [32].

## 4. Dietary Polyphenols Can Protect the Retina against Oxidative Stress and Its Consequences

Cranberry juice is known for its health-beneficial effects resulting from various pharmacological and biological properties, including hypolipidemic, antibacterial, anti-inflammatory, antioxidative, and anticancer properties, and that is why it is sometimes called “superfruit” [33]. Cranberries are rich in vitamins and minerals, but they also contain phenolic compounds—hydroxycinnamic acid, caffeine, coumaric acid, and ferulic acid. It was shown that cranberry juice with verified phenolic content acted protectively in ARPE-19 cells under oxidative stress induced by blue light exposure [34].

To consider the influence of the structure of anthocyanins on their effects in the eye, Wang et al. studied the influence of anthocyanins with different aglycones isolated from blueberry, blackberry, and strawberry (pelargonidin-3-glucoside, C3g, delphinidin-3-glucoside, and malvidin-3-glucoside) on ROS production in ARPE-19 cells exposed to visible light [35]. C3g proved to be the most effective ROS scavengers among all the tested compounds. In addition, C3g downregulated VEGF and inhibited senescence induced by the light exposure. These results show that the presence of an orthohydroxyl group in the structure of C3g may be a deciding factor for its high biological activity—antioxidant, antiangiogenic, and antisenescent/aging activity.

Tannic acid (TA) is a water-soluble polyphenol found in various plants, tea, and coffee [36]. It is reported to induce health beneficial effects, including anticancer, antimutagenic, antioxidative, and anti-inflammatory influences [37]. It was observed that the production of interleukin 18 (IL-18) induced by UVB was reduced by TA in human keratinocytes [38]. TA was shown to inhibit the interaction between the chemoattractant CXCL12/SDF-1*α* (stromal cell-derived factor-1*α*) and its receptor CXCR4 resulting in inhibition of angiogenesis [39]. That inhibition of the CXCL12/CXCR4 pathway by TA also contributes to its anti-inflammatory properties. Given that inflammation and neovascularization can be important for AMD pathogenesis, these results justify the studies on the role of TA in AMD. Chou et al. showed that TA inhibited the production of IL-6 induced by UVB and phosphorylation of STAT3 (signal transducer and activator of transcription 3) and downregulated the expression of complement factor B (CFB) in ARPE-19 cells [40]. CFB activation can be involved in AMD pathogenesis [41]. It was concluded that the protective action of TA against UVB-induced damage in ARPE cells required inhibition of the IL-6/JAK2 (Janus kinase 2)/STAT3 signaling pathway and its resulting anti-inflammatory action.

It was shown that curcumin increased the viability of ARPE-19 cells, which were challenged with H_2_O_2_ to simulate aging [42]. These cells were prone to apoptosis, which was inhibited by curcumin in both early and late stages. Furthermore, curcumin upregulated the antiapoptotic BCL2 protein and downregulated the proapoptotic proteins BAX and caspase-3. The oxidative stress biomarkers, including MDA (malondialdehyde), SOD, and GSH, were upregulated (SOD, GSH) or downregulated (MDA) by curcumin. Although the model of aging cells applied by authors is interesting, it rather reflects the process of premature cellular senescence, which certainly is associated with both cellular and organismal aging.

We showed that quercetin protected ARPE-19 cells against stress induced by 4-hydroxynonenal (HNE), an end product of lipid peroxidation [43]. A decreased mRNA expression of proinflammatory interleukins IL-6 and IL-8 as well as monocyte chemoattractant protein 1 (MCP-1) was observed. Additionally, quercetin influenced the p38, ERK (extracellular signal-regulated kinase), MAPK (mitogen-activated protein kinase), and CREB (cAMP response element-binding protein) signaling in stress conditions. All these changes were associated with an increased viability of ARPE-19 cells in HNE-induced oxidative stress. Therefore, there may be several mechanisms of the protective action of quercetin against oxidative stress in the retina, and its anti-inflammatory action may be of special significance. We obtained similar results for two other natural polyphenols, fisetin and luteolin [44]. These compounds reduced the activation of MAPKs and CREB, but did not affect NF-*κ*B (nuclear factor kappa B) or SIRT1 (sirtuin 1) in their anti-inflammatory action.

Quercetin was shown to protect against hydrogen peroxide-induced oxidative stress by the reduction of the decrease in mitochondrial function and viability in RPE cells isolated from the eyes of a human donor obtained from an eye bank [45]. These changes were attributed to the inhibition of caspase-3 activity, protection from senescence assessed by inhibition of beta-galactosidase, and reduction of expression of caveolin-1, a scaffold cytoplasmic membrane protein. Quercetin did not affect the intracellular level of glutathione.

Several other in vitro works showed that quercetin could effectively ameliorate the consequences of oxidative stress in different regions of the eye, including RPE cells [46–49]. Cao et al. studied the protective effect of quercetin in RPE cells both in vitro and in vivo. They showed that quercetin changed the transcription of many genes involved in apoptosis regulation, including the BCL2 (B-cell CLL/lymphoma 2), BAX (BCL2-associated X), FADD (Fas associated via death domain), CASPASE-3, and CASPASE-9 genes, in ARPE-19 cells facing oxidative stress induced by hydrogen peroxide [50]. In their in vivo research, Cao et al. used mice with double knockout (dKO) in the Ccl2 (C-C motif chemokine ligand 2) and Cx3cr1 (C-X3-C motif chemokine receptor 1) genes, which had acquired retinal injuries similar to those observed in AMD. Fundus and histological examinations did not show that quercetin slowed the progression of AMD-typical changes in dKO mice. However, quercetin-treated animals showed decreased serum levels of nitric oxide, decreased NADP+/NADPH ratios, and increased PGE-2 (prostaglandin E2) levels and COX (prostaglandin-endoperoxide synthase) activity, which suggest a systemic antioxidant action of quercetin. dKO mice showed an increased ocular mRNA expression of some proapoptotic genes and a decreased level of certain antiapoptotic proteins as compared to wild-type animals, but quercetin did not inhibit the elevated expression of proapoptotic proteins. It did not lower the increased transcription of proinflammatory mediators in the eyes of the dKO mice. This important work clearly shows that the observed protective action of quercetin in vivo can be substantially different from that expected on the basis of in vitro experiments. This work does not provide a clear explanation of these differences, but many factors can play a role, including the suitability of the animal model of AMD and relationship between in vitro and in vivo quercetin concentrations. Another problem is the accumulation of quercetin in ocular tissues.

It was shown that (-)epigallocatechin gallate (EGCG), an active phenolic component of green tea, protected ARPE-19 cells from oxidative stress by diminishing the production of hydrogen peroxide and activation of MAPK and COX-2 expression induced by UVA resulting in the general improvement of cell survival after UVA exposure [51].

A panel of vegetable polyphenols (bioflavonoids) containing EGCG, luteolin, apigenin, myricetin, quercetin, and cyanidin was studied to determine their effects on cell viability and proliferation, secretion of VEGF (vascular endothelial growth factor), and apoptosis in RPE cells obtained from human donors within 48 h of their deaths [52]. In general, most compounds of the panel inhibited viability and proliferation of RPE cells as well as VEGF secretion. The decrease evoked by luteolin, apigenin, myricetin, and quercetin was associated with the induction of necrosis by these compounds. Myricetin induced the generation of free radicals and the activation of calpain and phospholipase A2 resulting in necrosis independent of caspase-3. This work performed on a unique material shows that polyphenolic compounds, considered generally as profitable, can induce various adverse effects and therefore they can be applied with caution. Furthermore, that work shows that different compounds of a single class can display diverse modes of action, which is especially important when a natural product, containing several such compounds, is considered to be used in therapy or prevention.

Using a rat model of light-induced retinal degeneration (LIRD), Mandal et al. showed that curcumin inhibited the activation of NF-*κ*B and expression of genes, whose products inhibit cellular inflammatory response and increased expression of oxidative stress-related genes resulting in protecting human retinal cells 661W and ARPE-19 against light-induced stress [53].

Several studies addressed the antioxidant effect of polyphenolics in the retinal ganglion cells (RGCs). These cells directly contact the brain. In several studies, polyphenols showed similar antioxidant effects and mechanisms underlying these effects as they did in RPE cells [54, 55].

## 5. Polyphenols Can Activate NRF2 and Phase II Enzymes

NRF2 (NFE2L2, nuclear factor, erythroid 2-like 2) is a nuclear factor controlling the transcription and induction of many genes with diverse functions, including antioxidant defense, detoxification, DNA damage response, and cellular waste clearing (reviewed in [56]).

**Figure 4:**
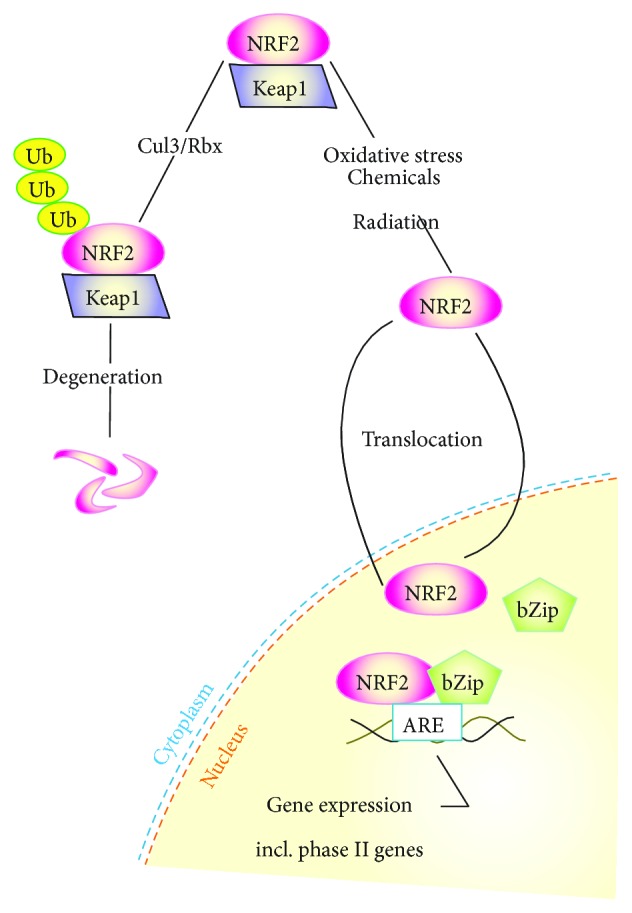
NRF2 (NFE2L2, nuclear factor, erythroid 2-like 2) activation. NRF2 in the cytoplasm is bound with its inhibitor, Keap1, and its amount is regulated by its ubiquitination and subsequent degradation. In stress condition, NRF2 is released from its inhibitor and translocates to the nucleus where it can transactivate many genes having the antioxidant response element (ARE), including phase II genes after complexing with a bZIP (basic leucine zipper) protein.

In normal conditions, NRF2 is degraded in the Cul3 (cullin-3)/Rbx1 (RING-box protein 1) E3 ubiquitin ligase-mediated pathway in the cytoplasm by its specific inhibitor, INRF2 (Keap1, Kelch-like ECH-associated protein 1) (Figure 4). Many chemicals, including polyphenols, antagonize the INRF2/NRF2 interaction inducing NRF2 activation and its nuclear export [57]. NRF2, after forming a heterodimer with a bZIP (basic leucine zipper) protein, controls the transcription of phase II enzymes, which are a major class of proteins protecting cells against oxidative stress [58]. Food polyphenols are reported to activate NRF2, which can, at least in part, explain the beneficial effects of high dietary intake of these compounds in preventing age-related diseases (reviewed in [59]).

HO-1 (heme oxygenase 1) is the major enzyme of heme catabolism degrading heme to carbon monoxide (CO), free Fe^2+^, and biliverdin, which is changed into bilirubin by biliverdin reductase. To limit ROS production in the Fenton reaction catalyzed by Fe^2+^ ions, they are bound to ferritin and exported by the Fe^2+^ pump. CO inhibits the production of cytokines and platelet aggregation and mediates vasodilatation, which can collectively inhibit AMD development. Therefore, HO-1 activation is a significant protective step in the retinal reaction to oxidative stress, which can be important for AMD induction and progression.

Cellular antioxidant mechanisms rely on the interaction of many proteins, including these with antioxidant response elements (AREs) in the promoters of their genes. These genes are under the transcriptional control of the NRF2 transcription factor [56]. It was shown that quercetin, fisetin, and eriodictyol induced the expression of NRF2 along with HO-1, a phase II protein [60]. In similar research, a purified bilberry extract containing anthocyanins or nonanthocyanin phenolics reduced the extent of ROS induced by the exposure of ARPE-19 cells to H_2_O_2_, and upregulation of the antioxidant enzymes, HO-1 and glutathione transferase (GSH), which was observed in that work, could be at least in part responsible for that ROS reduction [61]. Another phase II enzyme, NAD(P)H:quinone reductase 1 (NQO1) was reported to be upregulated by eriodictyol [62]. The expression of NRF2 was also reported to be modulated by EGCG from tea in human retinal pigmented cells exposed to methylglyoxal, a major precursor of advanced glycation end product [63].

NRF2 seems to be mainly responsible for the protective effect of taxifolin, a flavonoid common in citrus fruits, grapes, onions, and olive oil, against H_2_O_2_-induced oxidative stress in ARPE-19 cells. Taxifolin evoked the upregulation of NRF2, its translocation to the nucleus, and the induction of phase II enzymes: NQO1, HO-1, and glutamate-cysteine ligase modifier (GCLM) and catalytic (GCLC) subunits. Hanneken et al. reported that many dietary and synthetic flavonoids, including quercetin, baicalein, galangin, eriodictyol, and EGCG, protected against oxidative stress induced by hydrogen peroxide or tert-butyl hydroperoxide (t-BOOH) [60]. In these studies, quercetin displayed higher protective effects against death of ARPE-19 cells than vitamin E (tocopherol or its derivatives) and vitamin C (ascorbic acid). These authors stated that the observed effects could be mechanistically underlined by the upregulation of NRF2 and one of its downstream protein HO-1. However, no completely mutual relationship between protection from oxidative stress and NRF2/HO-1 upregulation was observed; i.e., some flavonoids that increased cell viability did not upregulate either NRF2 or HO-1. In addition, it is not clear why these authors observed almost 10-fold more effective protective action of ascorbic acid against cell death induced by t-BOOH compared with H_2_O_2_. Thermodynamical advantages of the interaction of vitamin C with t-BOOH over H_2_O_2_ seem not to be entirely responsible for such a high difference.

We did not observe upregulation of the NRF2 gene in ARPE-19 cells by pinosylvin (PS), a resveratrol analog occurring in Pinus species, although PS protected the cells against oxidative stress induced by hydroquinone [64]. Moreover, HO-1 was upregulated on PS treatment. Similar effect, i.e., protection from oxidative stress with increased HO-1 expression and no changes with NRF2, was obtained by Ishikado et al. with the extract of willow bark [65].

Curcumin was reported to protect ARPE-19 cells against hydrogen peroxide-induced oxidative stress by reducing ROS levels and upregulating HO-1 [66]. Curcumin inhibited apoptosis induced by oxidative stress, but only in the presence of HO-1 and not when the cells were transfected with siRNA for HO-1. Therefore, curcumin decreased detrimental consequences of oxidative stress in RPE cells through its interaction with HO-1, which is a necessary intermediate of the protective effect of this polyphenol in retinal cells. It was suggested that p38 activation might play a role in the activation of HO-1 by curcumin in RPE cells.

Li et al. showed that the protective effect of the curcumin analog 1,5-bis(2-trifluoromethylphenyl)-1,4-pentadien-3-one (C3) and curcumin itself against the cytotoxicity of acrolein, a compound present in cigarettes, in ARPE-19 cells dependent on the activation of NRF2 [67]. C3 displayed a much more effective protection than its parent compound. Both polyphenols ameliorate mitochondrial function impaired by acrolein. Moreover, both polyphenols activated the PI3/Akt pathway, but the NRF2 activation was independent of this effect and it was suggested that both C3 and curcumin directly disturb the NRF2/Keap1 interaction resulting in the release of NRF2 and its nuclear translocation with subsequent activation of phase II enzymes. This important work not only confirms the general mechanism of the protective effects exerted by different polyphenols—NRF2 activation and induction of phase II enzymes—but also shows that a subtle chemical modification of natural polyphenol may result in a significant increase in its profitable properties.

## 6. Dietary Polyphenols and Cellular Waste Clearing in AMD

AMD is featured by the accumulation of polypeptides, lipids, and/or RNAs, which are not completely cleared by the cellular waste clearing systems. These systems include ubiquitin-mediated proteasomal degradation, autophagy/mitophagy/heterophagy, and exocytosis [68].

AMD and some other eye diseases are featured by the age-related accumulation of the diretinal fluorophore A2E along with other members of the photoreactive retinaldehyde-derived molecule family. A2E can undergo photooxidation, increasing oxidative stress. Therefore, loading of RPE cells with A2E can mimic the situation when such cells are subjected to damage from accumulated A2E, which is considered a pathway in AMD pathogenesis. Polyphenol components of the *Vaccinium uliginosum* L. (V.U.) extract containing flavonoids, anthocyanins, phenylpropanoids, and iridoids reduced A2E photooxidation-induced death of ARPE-19 cells [69]. These compounds exerted a protective effect in the retina of rats exposed to high-intensity light, inhibiting the loss of the outer nuclear layer and nuclei. In another studies, Wang et al. showed that quercetin and C3g reduced ROS level and promoted the viability of ARPE-19 cells which were deficient in endogenous lipofuscin and loaded with A2E [70]. Additionally, these compounds inhibited the production of adducts of methylglyoxal and reaction of glutathione with photooxidized A2E and decreased the expression of the gene encoding receptor for advanced glycation end products. These polyphenols also protected glutathione from the reaction with photooxidized A2E. Quercetin and cyanidin-3-glucoside (C3g) also exerted a protective effect against oxidative stress and its consequences in photoreceptor cells, inhibiting the formation of 4-hydroxynonenal, which can be released from PUFA undergoing peroxidation by ROS resulting from the reaction of all-trans-retinal with phosphatidylethanolamine producing bisretinoid photoreactive species, including A2PE. A2PE can be transferred to RPE cells, where it is hydrolyzed to A2E with the involvement of phospholipase D (PLD). In similar research, grape skin extracts protected ARPE-19 cells loaded with A2E from apoptosis induced by blue light [71]. In another study, an antiapoptotic effect of the *Curcuma longa* L. extract and its curcuminoids against cytotoxicity induced by blue light exposure in A2E-loaded ARPE-19 cells was reported [72].

In other work, Wang et al. showed that quercetin and chlorogenic acid (CA), abundant dietary polyphenols, reduced the levels of inflammatory cytokines, including interleukins IL-8 and IL-*β*, TNF*α*, COX-2, and inducible nitric oxide synthase (iNOS), in the retina of rabbits exposed to visible light [73]. These polyphenols reduced oxidative stress induced by the light as evidenced by a decrease in HNE, MDA, 3-nitrotyrosine, and 8-OHdG (8-oxo-2′-deoxyguanosine), a marker of oxidative DNA damage levels, and upregulated HO-1. Quercetin and CA decreased the levels of proapoptotic (BAX) proteins and increased the levels of antiapoptotic (BCL2) proteins. In addition, the polyphenols reduced the expression of the angiogenic factors VEGF and HIF-1*α* (hypoxia-inducible factor 1*α*). Finally, quercetin protected against light-induced reduction of the outer nuclear layer. These two important works of Wang et al. provide valuable information on the possible mechanism of the protective action of quercetin and other dietary polyphenols in the retina under light-induced oxidative stress. In general, that profitable action can be underlined by a direct scavenging of ROS and anti-inflammatory, antiapoptotic, and antiangiogenic effects as well as by an indirect decrease in A2E photooxidation, reduction of methylglyoxal levels, and inhibition of the receptor of advanced end glycation product upregulation. Quercetin and other polyphenols can also regulate the activity of antioxidant enzymes, including phase II enzymes.

Wogonin is a flavonoid present in the root of Scutellaria baicalensis Georgi reported to have antioxidant and anti-inflammatory properties and exert anticancer effects [74, 75]. This flavonoid improved the viability of ARPE-19 cells challenged with hydrogen peroxide [76]. In search for the mechanism of this antioxidant protection, it was noted that wogonin displayed an antiapoptotic effect and downregulated p-Akt, but not the total level of Akt. This led to the speculation that wogonin could exert a protective effect in RPE cells through the modulation of the PI3K (phosphatidylinositol-3 kinase)/Akt pathway, as H_2_O_2_ induces PI3K and activates Akt in RPE cells, but not in the presence of a PI3K inhibitor [77]. Akt activation increases RPE cell survival and thus may protect RPE cells against the consequences of oxidative stress.

Quercetin was reported to inhibit choroidal neovascularization (CNV) induced by laser irradiation in rabbit eyes both in vivo and in vitro [78]. This flavonoid improved choroidal blood flow and decreased the migration of human umbilical vein endothelial cells during wound healing. Therefore, quercetin can be considered to improve anti-VEGF therapy, which is the only effective treatment in wet AMD [79]. In that therapy, wet AMD patients receive a series of intravitreal anti-VEGF injections but there are reports of some adverse events and complications [80]. Bevacizumab (BEV, Avastin), one of the most commonly applied anti-VEGF drugs in wet AMD therapy, is not free from unwanted side effects [81]. In clinical practice, BEV neutralizes all VEGF secreted from RPE cells, which leads to the disturbance in RPE homeostasis as VEGF is essential for the maintenance of the retinal structure [82]. The combined action of BEV and resveratrol only partly neutralized the secreted VEGF in ARPE-19 cells as compared with the cells treated with BEV singly, when neutralization of all VEGF occurred [83]. An increase in RPE phagocytosis and a decrease in epithelial-mesenchymal transition (EMT) were observed in that study. EMT can be associated with profibrotic changes in RPE cells following BEV exposure [84]. It was concluded that inhibition of EMT by resveratrol was underlined by the activation of the Notch 4 signaling. On the other hand, increased phagocytosis in the presence of resveratrol is in line with our research showing autophagy induction by resveratrol [85]. Therefore, resveratrol has a potential to ameliorate the adverse effects of BEV therapy in wet AMD. Resveratrol is present in the skin of grapes, and its high concentration is found in red wine. Other sources of resveratrol are blueberries, raspberries, and mulberries. Resveratrol has been combined with vitamins and omega-3 unsaturated fatty acids and other substances on the basis of the AREDS recommendation into the Resvega formulation, and we showed that it induced autophagy in ARPE-19 cells [85].

Resveratrol was reported to protect against H_2_O_2_-induced oxidation and death of ARPE-19 cells [86]. However, when acting in nonstress condition, resveratrol inhibited cell proliferation associated with the inhibition of the MAPK/ERK1/2 pathway. Inhibition of RPE cell proliferation can be translated into the protective action of resveratrol against proliferative vitreoretinopathy (PVR), associated with hyperproliferation of RPE cells [87]. Although that work discusses the obtained results in the context of not only PVR but AMD as well, the problem of proliferation of retinal cells from the periphery to the center in the process of limited regeneration of damaged retinal regions was not addressed.

Resveratrol proved to be protective against oxidative stress in human RPE R-50 cells induced by acrolein singly or in combination with hydrogen peroxide [88]. Acrolein inhibited phagocytosis function of RPE cells, and resveratrol protected against this inhibition. Moreover, resveratrol showed a synergistic protective effect with zeaxanthin.

Xu et al. reported a strong protective effect of a mixture of 8 polyphenol (catechins) extracts of green tea (GTP) against stress induced by UVB irradiation in a human RPE cell line [89]. GTP increased the viability of UVB-irradiated RPE cells in a dose-dependent manner up to a concentration of 140 mg/l, but further increasing GTP concentration resulted in a decrease in cell viability. The protective effect was stronger when the cells were preincubated than when they were postincubated with GTP. UVB irradiation induced several abnormalities in the microstructure of RPE cells, including deformation to mitochondria, and GTP served both before and after UVB exposure alleviated these changes. DNA damage analysis, made by a simple agarose gel electrophoresis, showed a nonspecific DNA fragmentation by UVB and a protective influence of GTP on this effect. However, the authors' conclusion on stimulating DNA repair by GTP on the basis of these results is not fully justified. GTP attenuated the suppression of the expression of survivin, an apoptosis inhibitor and cell cycle regulator, but this effect could not be directly associated with the antiapoptotic action of GTP as neither was apoptosis studied in that work, nor was DNA damage specifically attributed to apoptotic fragmentation of DNA.

Chang et al. used human RPE cells obtained by differentiation of induced pluripotent stem cells created by reprogramming of T cells derived from a patient with dry AMD (AMD-RPEs) [90]. In general, these cells showed a reduced antioxidant capacity as compared with normal RPE cells. Preincubation with curcumin resulted in a decrease in ROS level and cell death induced by H_2_O_2_ incubation. Curcumin increases the expression of the antioxidant genes HO-1 and SOD2 (superoxide dismutase 2), which were downregulated in AMD-RPEs as compared with control RPE cells. On the other hand, AMD-RPEs showed lower levels of expression of the PDGF, VEGF, and IGFBP-2 genes and curcumin increased the levels of mRNA of all the three genes. Moreover, H_2_O_2_ induced the secretion of PDGF and VEGF and curcumin inhibited it. It was also observed that curcumin inhibited the JNK (Janus kinase) pathway, which is important in cellular death and survival [91]. Some other aspects of this important work will be discussed in the concluding section.

## 7. Conclusions and Perspectives

Dietary polyphenols are often reported to have beneficial effects on human health. One of such effects is the improvement of visual functions and protection of the eye by anthocyanins contained in blueberries. Polyphenolics participate in such fundamental for the vision process as the visual cycle. C3g was shown to inhibit the activation of the G-protein transducin by metarhodopsin II in the visual cycle [92]. The process of activation requires GTP-GDP exchange, and the effect of the inhibition by C3g might have been underlined by the inhibition of cyclic GMP phosphodiesterase by this anthocyanin [93]. The regeneration of rhodopsin in the retinoid cycle is a major process involved in the visual signal transduction [94]. It was reported that anthocyanins present in blackcurrants, including C3g, accelerated the regeneration of rhodopsin [6]. Effects of polyphenolics on the visual cycle were reviewed by Kalt et al. [3].

There is a rich literature on the effects of polyphenolics on the retina, both in vitro and in vivo, and this review does not pretend to be complete. Moreover, it intentionally limits the effects to those associated with NRF2 activation and cellular waste clearing, apart from presenting their general antioxidant action. However, it can be concluded that some, if not all, dietary polyphenols exert a multipathway action in the eye, so they cannot be simply classified due to their specific mechanisms of action.

Most of the studies considered in this review were performed in vitro on cells to model AMD pathogenesis. The problem of the suitability of such models is out of the scope of this review. However, these in vitro studies were performed mostly with chemically pure polyphenols or their extracts from plants. Therefore, their dietary recommendations to prevent AMD or slow its progression should rather concern diet supplements, but not natural products, containing many different chemical substances, including other polyphenols, which can significantly modify the action of a particular compound. This can be the main source of the differences between results of in vivo research with polyphenol concentrations established on the basis of in vitro studies, as shown by Cao et al. [50]. Limiting of bioavailability of polyphenols by other substances can significantly contribute to this difference as shown for quercetin [95]. In this context, important are the results of Reboul et al. who showed that a mixture of polyphenols (gallic acid, caffeic acid, (+)-catechin, and naringenin) reduced lutein uptake in Caco-2 TC-7 colon cancer cells [96]. These studies were inspired by the authors' observation that the addition of a mixture of antioxidants, containing polyphenols, to a lutein-containing meal decreased the postprandial lutein response in the chylomicron-rich fraction in healthy men.

In general, AMD is an incurable disease and only its wet form can be targeted in anti-VEGF therapy with intermediary success, and efforts are made to increase the efficacy and decrease the side effects of the therapy. Resveratrol and its analogs proved to be promising agents in that regard, so further studies with other polyphenols, both natural and synthetic, can improve the anti-VEGF therapy in wet AMD [83, 97–99].

Any study addressing the dietary intervention in AMD, including polyphenolics, should take into account the genetic susceptibility to this disease—as it was done in some clinical trials on dietary supplementation in AMD. However, genetic constitution also influences the metabolism of polyphenolics. Besides, gut microbiota is especially important in the fate of ingested polyphenols, especially when they are provided in natural products. The epigenetic pattern, an important element in gene expression, can be modified by nutrition. It may also influence the effect of nutrition. Of course, epigenetic modifications constitute a panel consisting of many variants of DNA methylation, chemical modifications of histones, miRNA, and lncRNA, and it is unlikely to comprehensively provide the actual epigenetic status of an individual to adjust general dietary recommendations.

**Figure 5:**
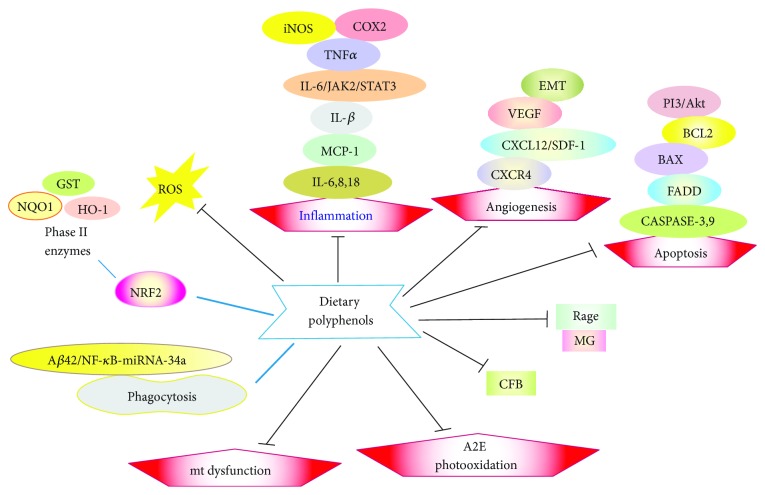
Modes of action of dietary polyphenols in the prevention of age-related macular degeneration. Full names of proteins and other details are in the main text.

Direct scavenging of ROS by dietary polyphenols as the most universal and spectacular action of these compounds is of special interest. However, only few studies addressed this issue by a comparison of results obtained with preincubation with phenolic compounds with and without their washing out before the action of a stress-inducing factor, usually a peroxide or light to avoid any physical contact between a phenolic and the oxidant. Apart from a direct ROS scavenging, many others including antioxidative mechanisms can underline the beneficial effects of polyphenols in AMD (Figure 5). Further studies are needed to precisely describe all these and other pathways which can be important for the action of these compounds in AMD. Such studies should pay attention to the structure-function relationship, as a subtle change in the polyphenol structure can substantially change its properties.

Most of the evidence about the influence of dietary polyphenols on the progression of AMD came from in vitro and in vivo animal studies. Therefore, prospective interventional studies are needed to confirm these findings.
